# Discounting and Augmentation in Causal Conditional Reasoning: Causal Models or Shallow Encoding?

**DOI:** 10.1371/journal.pone.0167741

**Published:** 2016-12-28

**Authors:** Simon Hall, Nilufa Ali, Nick Chater, Mike Oaksford

**Affiliations:** 1 Department of Psychological Sciences, Birkbeck College, University of London, London, United Kingdom; 2 Department of Psychology, Southampton Solent University, Southampton, United Kingdom; 3 Behavioural Sciences Group, Warwick Business School, Warwick University, Coventry, United Kingdom; Southwest University, CHINA

## Abstract

Recent research comparing mental models theory and causal Bayes nets for their ability to account for discounting and augmentation inferences in causal conditional reasoning had some limitations. One of the experiments used an ordinal scale and multiple items and analysed the data by subjects and items. This procedure can create a variety of problems that can be resolved by using an appropriate cumulative link function mixed models approach in which items are treated as random effects. Experiment 1 replicated this earlier experiment and analysed the results using appropriate data analytic techniques. Although successfully replicating earlier research, the pattern of results could be explained by a much simpler “shallow encoding” hypothesis. Experiment 2 introduced a manipulation to critically test this hypothesis. The results favoured the causal Bayes nets predictions and not shallow encoding and were not consistent with mental models theory. Experiment 1 provided qualified support for the causal Bayes net approach using appropriate statistics because it also replicated the failure to observe one of the predicted main effects. Experiment 2 discounted one plausible explanation for this failure. While within the limited goals that were set for these experiments they were successful, more research is required to account for the pattern of findings using this paradigm.

## Introduction

Conditionals, which are typically rendered in English as *if p then q* (where *p* is called the *antecedent* and *q* is called the *consequent*), are essential to human inference. Conditional sentences are used to express a variety of relations, such as causation (*if you turn the key*, *then the car starts*), deontic regulations (*if you are drinking beer*, *you must be over 18*), and property attribution (*if it’s a raven*, *then its black*). Conditionals allow us to think hypothetically about what would (or should) happen in the world should certain conditions expressed in the antecedent, *p*, obtain. Conditionals therefore also underpin much of our decision-making. Much of what we know about reasoning with conditionals has come from the investigation of causal conditionals. That is, conditionals like *if you turn the key the car starts*, where the antecedent is the cause of the effect described in the consequent. Such reasoning does not seem to be accounted for well by the material conditional of standard logic, in which *if p then q* is true if *p* is false or *q* is true, otherwise it is false. This situation has led to the development of psychological theories that deviate more or less from standard logic. The two main theories in this area are mental models theory [1, 2, 3] and the new paradigm, probabilistic approach [4, 5, 6, 7, 8].

Recently, these two theories were compared for their ability to account for discounting and augmentation inferences with causal conditionals [9, 10, 11, 12]. Discounting occurs when there are two possible causes of an effect. So, if you know the lights went out and then discover that there was a power cut this discounts the fuse blowing as the cause of the lights going out. Augmentation occurs when two effects have a common cause. So, if you don’t know whether someone has chickenpox, knowing that they have spots increases the likelihood that they have a fever because these effects are correlated by their common cause, having chickenpox. The evidence from two experiments [9] was most consistent with the probabilistic approach. This approach appealed to causal model theory [13, 14, 15] in which it is assumed that causal Bayes nets [16, 17, 18, 19] provide the mental representations that underpin causal conditional reasoning.

In the experiments we report here we first resolve some of the limitations of these experiments [9]. The analysis of the data was inadequate on two levels. Multiple items were used and analyses by participants and items reported. However, this approach does not avoid the problem of language as a fixed effect fallacy [20]. Such data is better analysed using appropriate mixed effect models [21] where both participants and items can be treated as random effects. Moreover, the response variable in Experiment 2 in [9] was on an ordinal three point scale but the data were averaged over items and analysed assuming a continuous ratio scale. Both of these limitations argue that the results of these previous experiments may be artifacts of using inappropriate data analytic methods.

We have two goals for the experiments we report in this paper. First, we attempt to replicate the results in [9] comparing causal models and mental models theories using appropriate data analytic methods. In particular, we replicate their Experiment 2 and analyse the data using more appropriate cumulative link function mixed effects models [22, 23]. Second, the results of our first experiment and Experiment 2 in [9] could be explained by what we call the “shallow encoding hypothesis” which we discuss in the introduction to our second experiment. This experiment introduces a critical manipulation that may distinguish this hypothesis from causal model theory.

We first introduce causal model theory as it applies to conditional reasoning and introduce the predictions it makes for the experimental paradigm used in [9]. We then do the same for mental models theory. We then introduce Experiment 1 which replicates Experiment 2 in [9] and analyses the date using cumulative link function mixed effects models.

### Causal Model Theory

The two examples we used to introduce discounting and augmentation can be expressed using pairs of conditional sentences. For discounting the conditionals are:

$$\begin{array}{l} If\;the\;fuse\;blows\;\left(p\right)\;then\;the\;lights\;go\;out\;\left(q\right)\\ If\;there\;is\;a\;power\;cut\;\left(r\right)\;then\;the\;lights\;go\;out\;\left(q\right) \end{array}$$ (A)

For augmentation the conditionals are:

$$\begin{array}{l} If\;someone\;has\;fever\;\left(p\right)\;then\;they\;have\;chickenpox\;\left(q\right)\\ If\;someone\;has\;spots\;\left(r\right)\;then\;they\;have\;chickenpox\;\left(q\right) \end{array}$$ (B)

In (A), the conditionals are causal, that is, *p* and *r* are the causes of the effect *q*. In contrast, in (B) the conditionals are diagnostic, that is, *p* and *r* are the effects of the cause *q*. It has been known for some time that there are profound differences between the causal (or predictive) and diagnostic cases [24]. This is clear for (A) and (B). According to deductive logic, they should produce similar inferences but the causal structures they suggest are very different. According to causal model theory, the pairs of conditionals in (A) and (B) are mentally represented as different causal Bayes nets [12, 13] that lead to different inference patterns not predictable by deductive logic alone.

**Fig 1:**
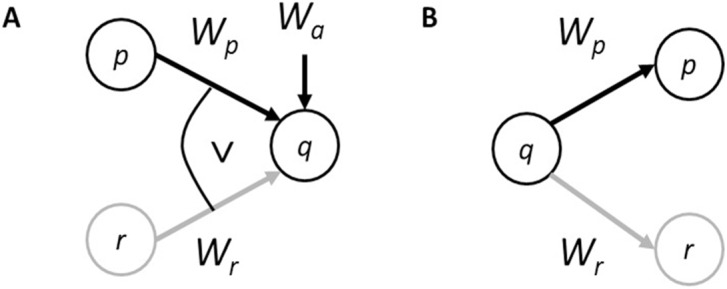
A. Common effect structure with a noisy-OR integration rule. B. Common cause structure.

Causal Bayes nets (CBNs) treat the causal dependencies that people believe to be operative in the world as basic [16, 17]. These dependencies are represented as edges in a directed acyclic graph (see Fig 1). The nodes represent Bayesian random variables. They also represent the relevant causes and effects, with the arrows running from cause to effect, i.e., the arrows represent causal direction. Nodes that are not connected represent variables which are conditionally independent of each other. The *parents* of a node are those that connect to it further back down the causal chain. These networks have probability distributions defined over them that partly rely on the dependency structure. So for example, in Fig 1A, the joint distribution over the three variables, *Pr*(*p*, *q*, *r*) = *Pr*(*p*)*Pr*(*r*)(*q*|*p*, *r*), whereas in Fig 1B it is *Pr*(*q*)*Pr*(*p|q*)(*r*|*q*).

Integration rules determine how the multiple parents of a node combine, e.g., the noisy-OR rule (see, Fig 1A). Suppose, in Fig 1A *p* and *r* represent *the fuses blowing* and *power cut* respectively as in (A). These are independent causes of *the lights going out* (i.e., *q*). The probability of the lights going out is,

$$Pr(q=1|p,\;r)=1\;–\;\left(1\;–\;W_{a}\right)\Pi_{i=p,r}{\left(1-W_{i}\right)}^{ind\left(i\right)}$$ (1)

Where, for example, *ind*(*p*) = 1 if the fuses blow and 0 if they do not. *W*_*i*_ is the probability of *q* given cause *i* (in the absence of alternative causes *a*). Thus, the weights, *W*_*i*_, are causal powers [25]. *W*_*a*_ is the weight attributed to alternative causes of *q*, other than *p* and *r*, which are assumed to be present in the causal background. If this were a deterministic system, *Wr* = *Wp* = 1, and there are no other causes of the lights going out (i.e., *W*_*a*_ = 0), then Eq 1 is equivalent to logical inclusive *OR*. It gives probability 1 unless both causes are absent when it gives probability 0, that is, if the fuses have not blown and there has not been a power cut, then the lights are on.

This view commits one to more than probability theory [16, 17]. A recent review [18] summarises the additional assumptions made in Bayes nets, which are mainly about making inference tractable. The most important assumption in this respect is the causal Markov property that causes "screen off" their effects so that inferences about any effect variable depend only on its direct causes and not on any of the other effects or indirect causes. For example, having chickenpox causes fever and spots. If it is known that someone has chickenpox then these effects are independent, i.e., manipulating one, e.g., using a cold compress to reduce fever, will not affect the other, the patient will still have spots. Moreover, if someone is known to have chickenpox these effects are independent of any of the causes of having chickenpox. While there are detractors of the Bayes net approach [26, 27], it is argued in [18, p. 111] “that the approach helps us to understand real causal systems and how ordinary people think about causality.” They may also help us to understand how people reason with conditionals.

The common effect structure in Fig 1A is the mental representation or interpretation of (A). The common cause structure in Fig 1B is the mental representation or interpretation of (B). All of the predictions for these experiments rely only on the qualitative pattern of independence relations embodied in the structures of the networks depicted in these figures. For the common effect structure (Fig 1A), the causes *p* and *r* are independent, i.e., *Pr*(*p|r*) = *Pr*(*p*), because *p* and *r* have no common parent. So, the probability of the fuses blowing (*p*) is independent of power cuts (*r*). However, they are not independent if the effect is known to have occurred, i.e., *Pr*(*p|r*, *q*) ≠ *Pr*(*p|q*). Indeed, it is easily proven that *Pr*(*p|r*, *q*) < *Pr*(*p|q*) [28]. That is, if it is known that the lights went out, then the probability that the fuses blew is less when it also is known that there was a power cut. Consequently, discounting is predicted. For the common cause structure (Fig 1B), the effects *p* and *r* are independent given *q*, i.e., *Pr*(*p|r*, *q*) = *Pr*(*p|q*). That is, given it is known that someone has chicken pox then the probability of having a fever (*p*) is independent of having spots (*r*). However, they are not independent if the cause is not known to have occurred, i.e., *Pr*(*p|r*) ≠ *Pr*(*p*). Indeed, it is easily proven that *Pr*(*p|r*) > *Pr*(*p*) [28]. That is, if it is not known whether someone has chicken pox, then the probability that they have a fever is greater when it is known that they have spots. Consequently, augmentation is predicted.

In [9], participants were presented with pairs of conditional sentences like (A) and (B). For the common effect structure ((A) and Fig 1A), using causal conditionals (CE), they were then presented with the following information:

 **CEC** ***q* Present (C)**    **CENC** ***q* Absent (NC)**

    The lights go out (*q*).     There is a power cut (*r*)

    There is a power cut (*r*)

In Experiment 2 in [9], after being told that there was a power cut participants were asked whether this fact decreases, increases, or has no effect on the likelihood that the fuse has blown (*r*). According to causal model theory, in the consequent (here the effect) present condition (C), participants should say the probability that the fuse has blown decreases. Whereas in the consequent absent condition, participants should say that there is no change. For the common cause structure ((B) and Fig 1B), using diagnostic conditionals (EC), participants were then presented with the following information:

  **ECC** ***q* Present (C)**      **ECNC** ***q* Absent (NC)**

    They have chickenpox (*q*).     They have spots (*r*)

    They have spots (*r*)

According to causal model theory, in the consequent (here the cause) present condition, participants should say the presence of spots has no effect on the probability they have a fever (*p*). Whereas in the consequent absent condition, participants should say that this probability increases. We now derive predictions for the mental models theory.

### Mental Models

Mental models represent the possible states of affairs in the world that are admitted by the truth of a conditional statement. These correspond directly to the rows of a truth table in which the conditional is true. Previous work in mental models on *naive* probabilities assumed that each truth table case was equally probable [29]. The same assumption was made in deriving predictions in [9].

A *p* *q* .25  B *p* *q* .33  (3)

  *p* *¬q* .25   ¬*p* *q* .33

  ¬*p*  *q* .25   ¬*p* *¬q* .33

  ¬*p* *¬q* .25

(3) shows the consequences of this assumption (“¬” = not). (3A) represents all four possible true and false arrangements of two propositions, *fuse blows* (*p*) and *lights go out* (*q*). These are assumed to be equiprobable. Each possibility is therefore assigned a probability of .25. On learning that, *if p then q* is true, the case where the conditional is false is ruled out. That is, the case in which the *fuse blows* is true (*p*) and *the light go out* is false (¬*q*) is no longer possible. This results in the mental model in (3B). With this case removed the probability mass has to be redistributed maintaining equiprobability. For the modus ponens inference, the categorical premise asserts that the *fuse blows* is true. This further rules out the bottom two possibilities in (3B). Therefore, the probability mass all moves to the first row. As this case is now the only possibility, *the lights go out* must be true, i.e., Pr(*q*) = 1.

### Mental Models, Discounting and Augmentation

According to mental models theory, people recode (A) as *if the fuse blows* (*p*) OR *there is a power cut* (*r*), *then the lights go out* (*q*) [30]. People, therefore, construct the mental model in (1').

*Fuse blowing* *Power cut* *Lights off* (1')

*Fuse blowing* *¬Power cut* *Lights off*

*¬Fuse blowing* *Power cut* *Lights off*

*¬Fuse blowing* *¬Power cut* *Lights off*

*¬Fuse blowing* *¬Power cut* *¬Lights off*

In (1′), the left to right ordering represents the cause to effect temporal order. In mental models theory, (B) is recoded as: *if someone has chicken pox* (*q*), *they have a fever* (*p*) AND *spots* (*r*) [31]. People, therefore, construct the mental model in (2′).

*Chicken Pox* *Fever* *Spots* (2')

*¬Chicken Pox* *Fever* *Spots*

*¬Chicken Pox* *Fever* *¬Spots*

*¬Chicken Pox* *¬Fever* *Spots*

*¬Chicken Pox* *¬Fever* *¬Spots*

Deriving predictions proceeds as follows (assuming equiprobability). Take (1′) for the consequent absent condition, *Pr*(*r*) is the proportion of models in which *r* is true. That is, those where *there is a power cut* is true divided by the number of models, i.e., 2/5. *Pr*(*r*|*p*) requires excluding all the models in which *p* (*fuse blowing*) is false and re-calculating the proportion of models in which *r* is true in the remaining models, i.e., 1/2. The degree of discounting or augmentation is the difference, that is, 1/2 − 2/5 = 1/10. So augmentation is predicted. Consequently, when it is not known whether the lights go out mental models theory predicts that people should say that the probability that there was a power cut increases after they learn that the fuse blew. This prediction contrasts with causal model theory, which predicts no change. If we do the same calculation for the consequent (i.e., the effect) present condition, then *Pr*(*r*|*q*) = *½* *and Pr*(*r*|*p*,*q*) = *½*. That is, there is no difference. Consequently, when it is known that the lights go out, mental models theory predicts that people should say there is no change in the probability that there was a power cut after they learn that the fuse blew. This prediction contrasts with causal model theory, which predicts discounting,

Now take (2′) for the consequent absent condition. *Pr*(*r*) is the proportion of models in which *has spots* is true divided by the number of models, i.e., 3/5. *Pr*(*r*|*p*) requires excluding all the models in which *p* (*has fever*) is false and re-calculating the proportion of models in which *r* is true in the remaining models, i.e., 2/3. The degree of discounting or augmentation is the difference, that is, 2/3 − 3/5 = 1/15. So, augmentation is predicted. Consequently, when it is not known whether someone has chickenpox, mental models theory predicts that people should say that the probability that someone has spots increases after they learn that she has a fever. This prediction is consistent with causal model theory. If we do the same calculation for the consequent (i.e., the cause) present condition, then *Pr*(*r*|*q*) = 1 *and Pr*(*r*|*p*, *q*) = 1, i.e., there is no difference. Consequently, when it is known that someone has chickenpox, mental models theory predicts that people should say there is no change in the probability that someone has spots after they learn that she has a fever. This prediction is also consistent with causal model theory.

An important element of mental models theory is that people rarely represent the complete, or “fleshed out” mental models of some premises, as in (1′) and (2′). Rather they initially represent the premises in a reduced form, known as an *initial mental model*, which excludes the false antecedent models and does not show false cases. Consequently, the initial mental models of (1′) and (2′) are as follows:

$$\begin{array}{ccc} \left|\begin{array}{cc} Fuse\;blowing & Power\;cut\\ Fuse\;blowing & \\ & Power\;cut \end{array}\right| & \begin{array}{c} Lights\;off\\ Lights\;off\\ Lights\;off \end{array} & \begin{array}{c} \left(1\prime \prime \right) \end{array}\\ \begin{array}{cc} & \ldots \end{array} & & \end{array}$$

And,

$$\begin{array}{llll} \left|Chicken\;Pox\right| & Fever & Spots & \left(2\prime \prime \right)\\ & \ldots & & \end{array}$$

The ellipsis “…” indicates an *implicit* model containing the other possibilities which can be made explicit when fleshed out to produce the representations in (1′) and (2′). The brackets (“| |”) indicate that these true cases are exhausted and there are none in the implicit model. A great deal of the explanatory power of mental models theory derives from the postulation of these truncated initial representations. However, as argued [9], it is quite complex to incorporate them into simple calculations of naïve probabilities. In particular, how the implicit model and the possibilities it may contain figure in the calculation is problematic. We refer the reader to [9] where the predictions of initial mental models are clearly laid out: there should be no effects of whether the conditionals are causal (A) or diagnostic (B) or whether the consequent clause (*q*) is known or not.

It has recently been argued [32] that the application of the mental models theory of naive probability to discounting inferences in [9] was not faithful to the theory. The following citation identifies the issue [29, p. 68]: “Each model represents an equiprobable alternative unless individuals have knowledge or beliefs to the contrary, in which case they will assign different probabilities to different models.” We continue to compare causal model theory to the predictions of mental model theory with naïve probabilities for two reasons. First, we have previously argued that allowing different probabilities to be assigned to different models means there is little distinctive left in the mental models approach to probability [9, 33]. On this view, (1′) is simply the full joint probability distribution over *p*, *q*, and *r*, with the three missing cells of the contingency table assigned a probability of 0. But this is just to tacitly agree that naïve probabilities in mental models cannot account for simple discounting inferences and that a full probabilistic theory of inference is required.

Second, just allowing different probabilities to be assigned to different models does not allow mental models theory to *predict* discounting inferences. Only assigning just the right probabilities will achieve this. The probabilities that would need to be assigned are those that respect the independence constraints embodied in the causal model representations, e.g., the lack of direct connection between nodes *p* and *r* in Fig 1A depicts that they are independent. However, unlike causal models, mental models theory lacks the representational resources to express these constraints on these probabilities. This is analogous to logical languages which differ in expressibility. For example, propositional logic lacks the ability to express quantification (all, some) which requires predicate logic. That is, symbols and rules for the composition of quantifiers need to be added to propositional logic to express quantified claims. Similar principled additions would be required in mental models formalism to express the required independence relations. However, no such proposals have been forthcoming. In mental models, without the graphical representation of causes and how they constrain probability assignments, it is simply a matter of serendipity as to whether the right probabilities are attached to models.

### Predictions

**Fig 2:**
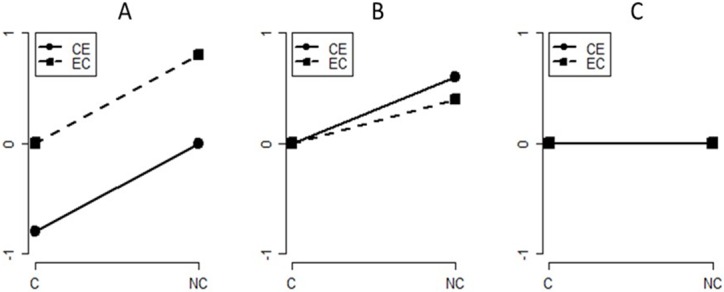
The predictions of the three theories: A: Causal Models (CM); B: Fully fleshed out mental models FM); C: Initial mental models (IM). The dashed lines with square markers are the predictions for the diagnostic conditionals (EC) and the full lines with circular markers are the predictions for causal conditionals (CE) when the consequent is present (C) and when it is absent (NC). *y* = 0 is where participants say there is no change, *y* = 1 is where participants say the likelihood goes up, *y* = -1 is where participants say the likelihood goes down, using the response procedure in Ali et al’s (2011) Experiment 2. Intermediate values are possible because of averaging over many items and participants.

The predictions of causal model theory (CM), fully fleshed out mental models (FM), and initial mental models (IM) are shown in Fig 2. In the rest of this paper, we will refer to the four conditions in the experiments using the abbreviations shown in this figure. So, for example, the causal conditional case when the consequent is known to have occurred will be designated CEC; the case when the consequent is not known to have occurred will be designated CENC; and similarly for the diagnostic (EC) rules.

In Experiment 1, we replicated Experiment 2 in [9]. We included a pre-test to evaluate the interpretation of the conditionals used and we used an appropriate cumulative link mixed models approach [22, 23] to analyse the data. The experimental hypotheses tested in Experiments 1 were as follows (derived directly from Fig 2):

1. According to CM, there should be main effects of both causal direction (causal (CE) vs. diagnostic (EC)) and consequent (present (C) vs. absent (NC)). According to FM, there should only be a main effect of consequent and according to IM there should be no effects of causal direction or consequent.
2. According to CM, but not FM or IM, for the CEC condition (causal direction, consequent present) the mean change rating should be less than in the ECC condition and below zero.
3. According to CM and FM, but not IM, for the ECNC condition (diagnostic direction, consequent absent) the mean change rating should be higher than in the CENC condition and above zero.

## Experiment 1

**Fig 3:**
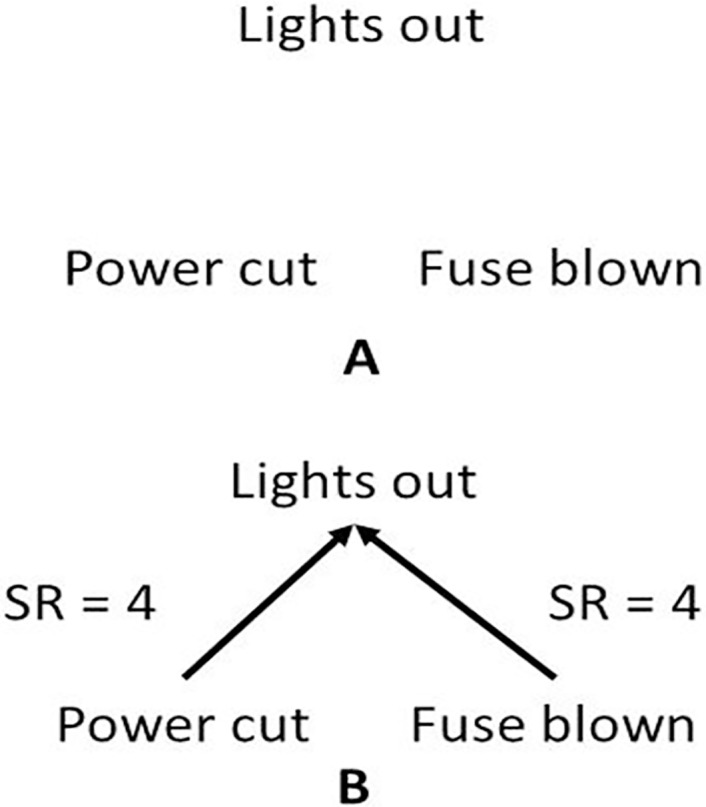
Materials for the pre-tests used in Experiments 1 and 2. Participants were shown the display in A and invited to draw in arrows for causal relations and rate the strength of the relation (SR) on a 1 to 4 scale, as in, for example, B.

This experiment replicated Experiment 2 in [9]. A separate group of participants (*N* = 18) also pre-tested the materials. They were shown the display in Fig 3A and were asked to draw in, using arrows, the appropriate causal connections, e.g., see Fig 3B. They then rated the strength of the causal connections they had drawn in as in Fig 3B. The pre-test data was used to select materials from an initial pool of 20 pairs of causal conditionals, as in (A), and 13 pairs of diagnostic conditionals, as in (B). We also embedded each pair of conditionals in a scenario, to render the inference participants were being asked to make more realistic and intuitive. Pairs of conditionals from the initial pool were excluded if:

1. The causal directions were not unidirectional.
2. For causal pairs (A), both causes failed to have a similar causal power, and for diagnostic pairs (B), the common cause failed to have a similar causal power for both effects.

### Method

#### Participants

These experiments were conducted in accordance with the Declaration of Helsinki and were approved by the local ethics committee of the Division of Psychology and Language Science, University College London. All participants provided written informed consent. No reward was offered. None had any prior knowledge of logic or of the psychology of reasoning.

In the pre-test phase, 18 undergraduate students (3 male, 15 female) from University College London volunteered to take part (mean age: 24.1 years, range: 18–53 years). These participants also pre-tested the materials used in Experiment 2. Participation in the pre-test excluded a participant from the experimental phases. For the main experimental phase, a further 40 undergraduate UCL students (5 male, 35 female) volunteered to take part (mean age: 20.5 years, range: 18 to 25 years).

The sample size was determined using prospective Bayesian power analysis [34, 35] and the results of Experiment 2 in [9]. A sample size that could lead to similar effect sizes to Ali et al (2011) was sought. In Experiment 2 in [9], the mode of the effect size for the CEC condition was -2.32 SD units [-3.30, -1.54] ([] = 95% HDI, i.e., Highest Density Interval); for the ECNC condition it was 2.76 SD units [1.91, 3.71]. The respective means were -.63[-.76, -.50] and .74 [.64, .85]. Simulated data were generated using the modes of the mean and SD for the CEC condition because the effect size was smaller. An *N* of 68 was used to simulate the data, which is the total number of participants in the experiments in [9]. A region of practical equivalence (ROPE) for the effect size was set to .75 SD units. This was set high so as to have sufficient power to detect large effects like those observed in [9]. The analysis showed that a sample size of 40 would provide a .93 (credible interval = .88 to .98) probability that the 95% HDI for the effect size would fall outside the ROPE.

#### Design

The experiment had two phases. In a pre-test phase, the initial pool of conditional statements was assessed. The main experimental phase was a 2 × 2 design with conditional (CE, EC) and consequent (C, NC) as within-subjects factors and with likelihood rating (see *Procedure*) as the dependent variable. There was a further phase where a single rating of the co-occurrence of *p* and *r*. We do not analyse these data here as they proved to be uninformative.

#### Materials

The materials (supporting information S1A and S1B Appendix) were pre-tested in paper booklets as shown in Fig 3A. The relevant causes and effects for the causal and diagnostic conditionals were randomly placed in the three locations shown in Fig 3. In the experimental phase, the conditionals were embedded in appropriate scenarios and were presented on PowerPoint. S1A and S1B Appendix show the pairs of conditionals that survived the exclusions based on the pre-test. The scenarios are available on request but an example causal scenario is as follows:

“You are meeting a friend in town and know he is planning to drive there. You know that:

If there is an accident on the main road, then he is caught in a traffic jam.

If there are road works on the main road, then he is caught in a traffic jam.

**While waiting for your friend to arrive you receive a text message saying he is caught in a traffic jam**.

**You wonder whether there is an accident on the main road**. (*Pr*(*p*|*q*))

**You now remember that there are road works on the main road**.

**Do you now think it is more, equally or less likely that there is an accident on the main road?** (*Pr*(*p*|*q*, *r*) >, =, < *Pr*(*p*|*q*)?)

*You arrive early and so do not know whether or not your friend is caught in a traffic jam*.

*However*, *you still wonder whether there is an accident on the main road*. (*Pr*(*p*))

*You now remember there are road works on the main road*.

*Do you now think it is more*, *equally or less likely that there is an accident on the main road*? (*Pr*(*p*|*r*) >, =, < *Pr*(*p*)?)

The text in bold is the consequent known condition (C). When the consequent was not known (NC) this text was replaced by the text in italics. As in Experiment 2 in [9], participants were asked for an ordinal *change rating* of whether *p* was “more likely” (+1), “equally likely” (0), or “less likely” (-1) after being told that *r* had occurred (the number in brackets shows the rating assigned to each category).

To provide as much variation as possible, within each condition, causal (CE) and diagnostic (EC), participants performed the consequent task (C) with different materials to the not-consequent task (NC).

#### Procedure

**Fig 4:**
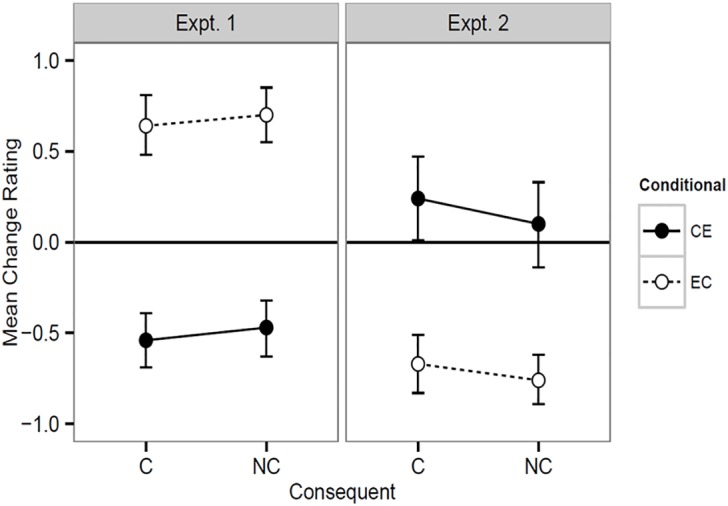
The results of Experiments 1 and 2. Expt. 1 (EC-AND, CE-OR), means and 95% confidence intervals (CIs) based on Model 3 in Table 1. Expt. 2 (EC-OR, CE-AND), means and 95% CIs based on Model 4 in Table 2.

In the pre-test, participants were asked to draw arrows from the statements that they thought were the *causes* to the statements that they thought were the *effects*. They were then shown an example like Fig 4B but without strength ratings. They were then asked to indicate how strong they thought the causal relationship was between a particular connection on a scale of 0–4; (0 for a *very weak* causal relation, 1 for *weak*, 2 for *average strength*, 3 for *strong* and 4 for *very strong*). One set of materials appeared per page of a booklet and the pages of each booklet were randomized. At the end of the pre-test, participants were debriefed.

In the main phase of the experiments, participants were tested individually. The scenarios were presented randomly on PowerPoint. Each scenario was presented on a separate coded slide. A score sheet was also provided with the code of each scenario alongside the three response options: "more likely", "equally likely" and "less likely". Participants circled their response. At the end of the Experiment, participants were thanked for their participation and debriefed as to the purpose of the experiment.

### Results and Discussion

#### Pre-test

The pre-test data was coded so that 0 = no causal relation, i.e., no causal link inserted into the diagram (see Fig 3). Consequently, the causal rating scale was rescaled to a six-point scale from 0 to 5. There were two target causal relations in each of the 33 pairs of conditionals tested. For CE these were *p* → *q* and *r* → *q* and for EC they were *q* → *p* and *q* → *r*. For each causal direction, there are four further possible non-target links, for CE: *q* → *p* and *q* → *r* and for EC: *p* → *q* and *r* → *q*. If the mean of the causal ratings for any of the non-target relations differed significantly from zero that pair of conditionals was excluded. In addition, if for any scenario the mean causal ratings differed between the target relations, that pair of conditionals was excluded. Standard *t-*tests were used, which allow the null to be rejected quite easily [36] and so are a conservative test option. If the null is rejected for any one of these analyses, that pair of conditionals was excluded. The exclusions left ten scenarios in the causal and seven in the diagnostic condition. Consequently, all materials retained after the pre-test had two unidirectional causal links and each pair was equally sufficient for their effect(s).

#### Main experiment

Fig 4 Panel A shows the results of Experiment 1 (for the raw data see supplementary material, S1 Experiment). Qualitatively they closely replicated the results of Experiment 2 in [9]. The prediction of augmentation for the ECNC condition and discounting for the CEC condition was confirmed. The pattern of errors was also replicated, that is, augmentation-like behaviour for the ECC condition and discounting-like behaviour for the CENC condition.

Our dependent measure, change rating (CR), is an ordinal variable (-1, 0, 1), so we analysed the data using cumulative link mixed models with a probit link function (function **clmm** in package **ordinal** implemented in R [22, 23]). We sequentially added participants and items as random effects. We compared models that corresponded to the predictions of CM, FM, and IM in Fig 2. These models are shown in Table 1. Model 1 corresponds to IM in which no effects of causal direction (CE or EC) or consequent (C or NC) are predicted. Model 1 is the null model which predicts the overall mean for all cells in the 2 × 2 design. Model 2 corresponds to CM in which there are main effects of both causal direction (CD) and consequent (C). For both Models 1 and 2, only a random intercept for participants was included. We assessed differences between models using the likelihood ratio and the Bayes factor. The Bayes factor (BF) is calculated using an approximation based on the Bayesian Information Criterion, BIC, that is, *BF* = e^(*BIC*(*Model* 1) − *BIC*(*Model* 2))^ [37]. Model 2 provided a better fit to the data than did Model 1, *G*^*2*^(2) = 371.57, *p* < .0001. The BF in favour of Model 2 is very high. We also show the AIC, Akaike Information Criterion, which is another index of fit that does not penalise a model for complexity (the number of parameters) as much as BIC. Model 3 also includes an intercept for items and the BF shows that this model was 5.3×10^11^ times more likely to have generated the data than Model 2 (*G*^*2*^(2) = 36.00, *p* < .0001). Consequently, there were random effects of items that needed to be taken into account. Random slopes for either participant or for items did not improve the fit. This is illustrated by Model 4 which produced a significantly better fit according to the likelihood ratio (*G*^*2*^(2) = 8.41, *p* < .001) but was less likely to have generated the data according to the Bayes factor (i.e., the BF in favour of Model 4 was less than 1). Model 5 corresponds to FM in Fig 2. FM, the fully fleshed out mental model, only predicts a main effect of consequent. We included random intercepts for participant and item and a random slope for participant because their inclusion led to significantly better fits for Model 4. Model 5 provided significantly poorer fits, *G*^*2*^(2) = 33.59, *p* < .0001, and it was 1.7×10^−14^ times less likely to have generated the data than Model 4.

**Table 1:** Cumulative link function models for Experiment 1.

Model	Pars	AIC	BIC	LR	df	BF
1. CR ~ 1 + (1|P)	3	1496.6	1510.2			
2. CR ~ CD + C + (1|P)	5	1129.2	1151.8	371.57	2	
3. CR ~ CD + C + (1|P) + (1|I)	6	1095.2	1122.3	36.00	1	5.3×10^11^
4. CR ~ CD + C + (1 + CD|P) + (1|I)	8	1090.8	1127.0	8.41	2	0.01
5. CR ~ C + (1 + CD|P) + (1|I)	7	1122.4	1154.0	33.59	1	1.7×10^−14^

Model 1 corresponds to IM (initial mental model); Model 5 corresponds to FM (fully fleshed out mental model); Model 2 corresponds to the CM (causal model) (see Fig 2). Models 2, 3, and 4 correspond to CM with differing assumptions about the structure in the random effects. CR = Change Rating; CD = causal direction; C = consequent. (1|*x*) = model includes an intercept for the random effect *x*, either P = participants or I = Items. (1 + CD|P) = model includes an intercept and a slope for causal direction for the random effect of participants. Pars = Number of parameters; AIC = Akaike Information Criterion; BIC = Bayesian Information Criterion; LR = Log Likelihood Ratio; df = degrees of freedom; BF = Bayes Factor. Each model is compared to the one above it in the list using the likelihood ratio and the Bayes Factor.

The results of these model comparisons showed that a model containing fixed main effects of causal direction and consequent, as predicted by CM, provides better fits to these data than the models predicted by FM and IM. However, Fig 4 also shows that the same pattern of errors occurred as in Experiment 2 in [9]. A model with just a fixed main effect of causal direction actually provides the best fit overall, that is, the predicted main effect of consequent was absent. The inclusion of interactions did not improve these fits. So, in terms of fitting the data, this experiment provides qualified support for CM (Hypothesis 1). Subsequent analyses and the graphs in Fig 4A are based on Model 3.

To test Hypotheses 2 and 3, we first used the R package **lsmeans** [38] to compute asymptotic statistics and significance levels for the simple effects comparisons for Model 3. We then used the estimated means and standard errors in *t*-tests for the comparisons to 0. Consistent with CM, but not FM or IM, for the CEC condition (causal direction, consequent present), the mean change rating was lower than in the ECC condition, *z*-ratio = 11.97, *p* < .0001 and less than zero, *t*(39) = 7.19, *d* = 2.30, *p* < .0001. Consistent with CM and FM but not IM, for the ECNC condition the mean change rating was higher than in the CENC condition, *z*-ratio = 12.02, *p* < .0001 and greater than zero, *t*(39) = 9.14, *d* = 2.93, *p* < .0001. However, consistent only with IM, there were no significant differences between the ECC and ECNC conditions, *z*-ratio = 1.38, *p* = 0.17, nor between the CEC and CENC conditions, *z*-ratio = 1.39, *p* = 0.17, although the trends were in the direction (ECNC > ECC; CENC > CEC) predicted by CM and FM. However, consistent with none of these theories, the change rating for the CENC was less than zero, *t*(39) = 5.97, *d* = 1.91, *p* < .0001, and for the ECC condition it was greater than zero, *p* < .0001, and ECC, *t*(39) = 7.69, *d* = 2.46, *p* < .0001.

These results replicated Experiment 2 in [9] using an appropriate cumulative link function mixed models approach. The results provide qualified support for causal model theory because it is the only theory that predicts a fixed main effect of causal direction, which was the dominant finding. In so far as neither FM nor IM predict the main effect of causal direction these results argue against the mental models account.

**Fig 5:**
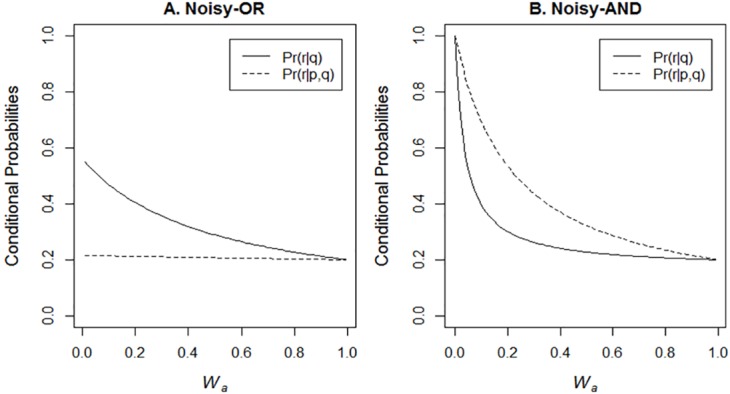
Discounting with the noisy-OR (A) and augmentation with the noisy-AND (B) integration functions varying the probability of alternative causes *W*_*a*_. For noisy-OR, unless *W_a_* = 1, *Pr*(*r*|*p*, *q*) < *Pr*(*r*|*q*) and discounting is predicted. For noisy-AND, unless *W_a_* = 1 *or* 0, *Pr*(*r*|*p*, *q*) > *Pr*(*r*|*q*) and augmentation is predicted. In these graphs *Pr*(*p*) = *Pr*(*r*) = .2 and *Wp* = *Wr* = *Wpr* = .9.

However, it could be argued that incorporating one of the assumptions discussed in [9] into the mental models account could provide at least a partial explanation for these findings. One condition that has been proposed to lead to discounting is that the causes *p* and *r* in Fig 1A are the sole causes of the effect [28]. If this were the case, then the lights could not be off in the absence of a power cut or the fuse blowing and so line four in the mental model in (1′) would be assigned probability zero and so would be excised. The revised mental model would predict a negative difference rating in the CEC condition and zero difference rating in the CENC condition as predicted by CM. There are three points to make. First, in [28] it is discussed at length what happens when *p* and *r* are not the sole causes of the effect. It is easily proved that discounting is still always predicted [28]. That is, in contradistinction to this ad hoc revision of mental models, discounting is still predicted in situations where line 4 in (1′) could not be assigned probability zero. The only essential condition whose violation can sometimes remove discounting is independence of causes, however many there may be [28]. Second, as Eq 1 and Fig 1A show, the noisy-OR integration function, which we used to introduce discounting in this paper, includes an explicit parameter for alternative causes other than *p* and *r*, i.e., *W*_*a*_. Fig 5A shows how *Pr*(*r*|*q*) and *Pr*(*r*|*p*, *q*) vary as a function of *W*_*a*_ and it reveals that discounting (*Pr*(*r*|*p*, *q*) < *Pr*(*r*|*q*)) is predicted as long as *W*_*a*_ < 1. This is in distinction to this proposal to allow mental models to predict discounting, which requires that *W*_*a*_ = 0, which is a much more restrictive condition. Third, to verify some claims about the materials in Experiment 2, we explicitly asked a separate group of participants to rate the probabilities of each effect given the absence of each cause for the materials in this experiment as well in Experiment 2 (see, supporting information: S1A Appendix). The probability of the effect given the absence of the cause was always nonzero (S1A Appendix reports, e.g., *Pr*(¬*q|*¬*p*), which were always less than 1). Consequently, for these materials, it would not seem that participants have adopted the assumption that this ad hoc revision of mental models theory requires to better explain the data.

As we have observed, the data only offered qualified support for CM. Moreover, as we mentioned in the introduction an alternative shallow encoding hypothesis may also explain this result. Experiment 2 was designed to provide a critical test of this hypothesis.

## Experiment 2: Shallow Encoding

Although previous results [9] were most consistent with causal models, there were some inconsistencies. In Experiment 1 and in Experiment 2 in [9], when it is not known whether the lights were off, participants still rated the power cut as less likely when they were told the fuse is blown (CENC). That is, they discounted when they should not have. Moreover, when they knew someone had chickenpox, they rated it more likely that they had spots given they have fever (ECC). That is, they augmented when they should not have. These errors seem to demonstrate a violation of the Markov condition that inferences about any effect variable depend only on its direct causes and not on any of the other effects or indirect causes.

Possible explanations concern associations between the antecedents, *p* and *r* [19] which we discuss further in the *Conclusion*. Here we explore a very simple explanation of these errors concerning the change rating response format used in the current Experiment 1 and in Experiment 2 in [9]. Taking the sentences in (A), participants are told that the lights go out and then that there is a power cut (CEC) or just that there is a power cut (CENC). In both conditions, they were asked whether the power cut increased or decreased the likelihood of the fuse blowing or left it the same. This response format imposes a memory load. In the CEC condition, for example, participants must interrogate their model assuming the lights are off and they have no information about a power cut and assess the probability that the fuse has blown. They then must interrogate the model again assuming there been a power cut and re-compute the probability of the fuse blowing. They must then compare this new result with the result they had to retain in working memory from the previous query. Even small memory loads like those imposed by easily visualizable materials can disrupt reasoning (see, [39] on the visual impedance effect).

A very simple strategy based on mental models theory could explain the results of Experiment 1. Mental models theory proposes that, due to working memory limitations, people frequently only construct partial representations of logical relations called “initial models” as we discussed in introducing Experiment 1. The results of Experiment 1 seem to indicate that people ignore the consequent manipulation. One reason for this could be that, because of the additional memory load imposed by the response format, they only formulate a partial representation of (A) and (B) which excludes the consequent. So for (A), which is recoded as *if the fuse blows* (*p*) OR *there is a power cut* (*r*), *then the lights go out* (*q*), the partial mental model *p* ¬*r*, ¬*p r* is constructed representing the possibilities allowed by an exclusive-or between *p* and *r*. On this reading, if *p* occurs *r* should not and if *r* occurs *p* should not. For (B), which is recoded as *if someone has chicken pox* (*q*), *they have a fever* (*p*) AND *spots* (*r*), the partial mental model *p r* is constructed representing the only possibility allowed by a conjunction between *p* and *r*. On this reading, if *p* occurs so should *r* and vice versa. This “shallow encoding” hypothesis predicts discounting in both the CEC *and* the CENC conditions and augmenting in both the ECNC *and* the ECC conditions, as we observed in Experiment 1.

Shallow encoding based on mental models is compatible with both mental models and a probabilistic approach based on CBN’s. It has recently been argued that mental models might provide the right kind of representation for recording the results of interrogating or sampling underlying probabilistic representations and that this might explain certain errors as people move from continuous to discrete representational formats [40, 41, 42]. This proposal means that some representational format like mental models would be required even if one takes a probabilistic view of the underlying deep logical structures over which people normally reason.

A critical test of the shallow encoding hypothesis would involve constructing materials with opposite linguistic recodings. That is, pairs of causal conditionals (CE) that can be recoded as *if p AND r*, *then q* and pairs of diagnostic conditionals (EC) that can be recoded as *if q*, *then p OR r*. If the shallow encoding hypothesis is true, then the results for the causal and diagnostic conditionals should be mirror images of the results of Experiment 1. So, there should be augmentation-like behaviour for the causal conditionals (CE) and discounting-like behaviour for the diagnostic conditionals (EC) independent of whether the consequent is known to have occurred.

Materials that implement this manipulation are (C) for the conjunctive antecedents and (D) for the disjunctive antecedents:

$$\begin{array}{l} If\;the\;plant\;is\;watered\;often\;\left(p\right),\;then\;it\;will\;grow\;\left(q\right)\\ If\;the\;plant\;receives\;light\;\left(r\right),\;then\;it\;will\;grow\;\left(q\right) \end{array}$$ (C)

$$\begin{array}{l} If\;a\;participant\;wears\;glasses\;\left(p\right),\;then\;their\;vision\;is\;poor\;\left(q\right)\;\\ If\;a\;person\;wears\;contact\;lenses\;\left(r\right),\;then\;their\;vision\;is\;poor\;\left(q\right) \end{array}$$ (D)

However, only materials like (C) are likely to prove discriminatory. (D) achieves an appropriate exclusive-OR reading, i.e., *if a person vision is poor*, *then they wear glasses OR contact lenses BUT NOT BOTH*. However, any implementation in a CBN would lead to the same conclusion that people should discount whether it is known the person’s vision is poor or not. The simplest implementation would be a two node network *q* ***→*** *C*, where *C* is a three level corrected vision variable with three mutually exclusive levels, *glasses* (*p*), *contacts* (*r*), and *nothing*. Such an implementation would make the same predictions as the shallow encoding hypothesis.

The materials in (C), however, could be discriminatory. Recently the CBN framework has been extended to conjunctive causes using a noisy-AND integration rule [35]:

$$Pr(q=1|p,\;r)\;=\;1\;–\;\left(1\;–\;W_{a}\right){\left(1\;–\;W_{pr}\right)}^{ind\left(p\right)ind\left(r\right)}$$ (2)

Using this rule in the common effect structure (Fig 1A) predicts augmentation when it is known that the plant grows (CEC) but not when this is not known (CENC). Knowing that the plant grows and is watered increases the probability that it also received light. Augmentation has been observed in a very similar condition [43]. Consequently, the predictions based on the noisy-AND rule contrast with the shallow encoding hypothesis in predicting no augmentation for the CENC condition.

So the contrastive predictions are that noisy-AND predicts less augmentation for the CENC condition in this experiment than for the ECNC condition in Experiment 1 but augmentation should be at similar levels for the CEC condition as for the ECC condition in Experiment 1. However, Eq 2 points to a manipulation that could allow the CEC condition to also discriminate between noisy-AND and the shallow encoding hypothesis. Fig 5B shows that augmentation reduces as *W*_*a*_ approaches either 0 or 1. At either extreme, no augmentation is predicted. A manipulation that should reduce the scope for alternative causes is when *p* and *r* are individually necessary for the effect. For example, plants will not grow in the absence of water or light, that is, there is no alternative cause that can make plants grow in the absence of these necessary causes. Using materials which encourage this interpretation should have the effect of reducing augmentation for the CEC condition. If this happens, then augmentation should be lower in a CEC condition in this experiment than in the ECC condition in Experiment 1.

4 According to both CM and shallow encoding, in the diagnostic direction (EC) change ratings should be lower than in the causal direction (CE) and below zero, that is, discounting-like behaviour should be observed.
5 According to CM, dependent on the manipulation of causal necessity, in the causal direction (CE), the change ratings should not differ from 0 for CENC but there should be some augmentation for CEC, that is, the change rating should be greater than 0.
6 Again dependent on the causal necessity manipulation, the levels of augmentation for CE in Experiment 2 should be much lower than for EC in Experiment 1 for both levels of consequent.

### Method

#### Participants

The sample size for the main experimental phase was smaller (*N* = 28) than in Experiment 1 but it was drawn from the same population, i.e., students at University College London. Sample size was set from a prospective power analysis as in Experiment 1 [34, 35]. CM predicts no (CENC) or less (CEC) augmentation for causal conditionals. According to shallow encoding, augmentation should be of a similar magnitude that that observed in Experiment 1 for the diagnostic/not-consequent condition. Consequently, a sample size was sought which could allow effect sizes of similar magnitude to Experiment 1 to be observed. Therefore, as in Experiment 1, the ROPE on the effect size was again set at .75 SD units. Simulated data were generated using the mean and SD for the diagnostic/not-consequent condition in Experiment 1 with an *N* of 108 which corresponds to the total number of participants in [9] and Experiment 1 where augmenting was observed. The prospective power analysis indicated that a sample size of 25 would provide a .93 probability that the 95% HDI for the effect size falls outside a .75 SD ROPE, i.e., a .93 probability that the null can be rejected. Consequently, an *N* of 28 provides sufficient power to detect an augmentation effect of this magnitude for the causal conditionals if there is one.

#### Design

The design of this experiment was the same as for Experiment 1 and the same participants carried out the pre-test for this experiment.

#### Materials

The materials are shown in supporting information S1C and S1D Appendix. In this experiment, for the CE-AND conditions materials were used to encourage an interpretation were the causes were individually necessary for their effects. This was to encourage a reduction in augmentation for the CEC condition [43]. Confirmation that the CE rule pairs were interpreted as individually necessary came from an additional on-line test using another 47 participants and two subsets of these materials (see S1A and S1C Appendix for the mean ratings for these subsets). Ratings were collected for *Pr*(*q*|*p*), Pr(*q*|*r*), *Pr*(*q*|¬*p*) and *Pr*(*q*|¬*r*) for these rule pairs in the CE conditions. As we were concerned with differences in necessity, *Pr*(*q*|¬*p*) and *Pr*(*q*|¬*r*) were subtracted from 1 and averaged. In the CE-AND condition in this experiment (*Pr*(*q*|*x*): $\bar{m}=.77$, SE = .03; *Pr*(¬*q*|¬*x*): $\bar{m}=.69$, SE = .03) causes were rated as less sufficient, *t*(13.33) = 2.99, *p* < .01, and more necessary, *t*(13.67) = 2.86, *p* < .01, than in the CE-OR condition (*Pr*(*q*|*x*): $\bar{m}=.89$, SE = .03; *Pr*(¬*q*|¬*x*): $\bar{m}=.57$, SE = .30) in Experiment 1. For three rule pairs (Rule pairs 2, 4, 5 in S1C Appendix) the cause was rated as more necessary than sufficient. We analysed the results in three ways, (i) using all the materials, (ii) restricting the CE-AND condition to just Rule pairs 2, 4, 5, and (iii) using just the subset of rule pairs for which we collected conditional probability ratings plus the restriction in (ii).

### Results and Discussion

#### Pre-test

The same exclusion criteria were used as in Experiment 1. The exclusions left six scenarios (from ten) for the causal conditionals and six (from 9) for the diagnostic conditionals.

#### Main experiment

Fig 4 Panel B shows the results of Experiment 2 (for the raw data see supplementary material, S2 Experiment). Qualitatively they closely follow the pattern predicted by CM. That is, in the CE causal direction there was no augmentation for CENC but some for CEC. Consistent with the shallow encoding and CM, in the EC direction there was discounting, regardless of whether the consequent was present (C) or absent (NC). We analysed the result in the three ways we mentioned at the end of the section *Materials*. However, which subsets of materials we used made no difference to the results. Consequently, unless stated the results using all materials are reported.

The cumulative link function models we compared are shown in Table 2. Model 1 is the null model. Model 2 corresponds to a model in which there is only a main effect of causal direction (CD). This model provides a better fit than the null model, *G*^*2*^(1) = 115.25, *p* < .0001. Adding a random effect of items, Model 3, improves the fit, *G*^*2*^(1) = 7.64, *p* < .01, and it is 6.17 times more likely to have generated the data than Model 2. Model 4 shows that adding a main effect of consequent (C) does not improve this fit. The fits were not improved by including either interactions (fixed effects) or slopes (random effects). According to the AIC, adding a main effect of consequent did not worsen the fit either. We, therefore, used Model 4 for the means shown in Fig 4 (Expt. 2) that we compared to test Hypotheses 4 and 5.

**Table 2:** Cumulative link function models for Experiment 2.

Model	Pars	AIC	BIC	LR	df	BF
1. CR ~ 1 + (1|P)	3	710.7	722.0			
2. CR ~ CD + (1|P)	4	597.3	612.6	117.30	1	
3. CR ~ CD + (1|P) + (1|I)	5	591.7	610.8	7.64	1	6.17
4. CR ~ CD + C + (1|P) + (1|I)	6	591.7	614.6	1.94	1	0.02

Model 1 is the baseline no effect model (i.e., the overall mean); Models 2, 3, and 4 correspond to CM with differing assumptions about the structure in the random effects. All acronyms are the same as in Table 1. Each model is compared to the one above it in the list using the likelihood ratio and the Bayes Factor.

Consistent with CM and the shallow encoding hypothesis, the mean change ratings for the ECC condition were lower than for the CEC condition, *z*-ratio = 7.05, *p* < .0001. The mean change ratings for the ECNC condition were also lower than for the CENC condition, *z*-ratio = 6.80, *p* < .0001. The change ratings were less than zero for the ECC condition, *t*(27) = 8.08, *d* = 3.11, *p* < .0001, and for the ECNC condition, *t*(27) = 10.72, *d* = 4.13, *p* < .0001. These results confirm Hypothesis 4. The change rating for the CEC condition was greater than zero, *t*(27) = 2.05, *d* = .79, *p* < .025 (one-tailed), but it did not differ from zero in the CENC condition, *t*(27) = .82, *d* = .32, *p* = .42. The Bonferroni correction for these multiple hypothesis tests mean that the significance level required for each individual hypothesis is .05/2 = .025. So the result for the CEC condition was just significant. However, the effect size was in the medium range (*d* = .79) and fell outside the ROPE (*d* = .75) we set in the power analysis to detect an augmentation effect. This was in contrast to the CENC condition which fell inside the ROPE. So we can have some confidence in this result for the CEC condition. Consequently, using materials for which the causes were individually necessary for the effect has reduced but not eliminated augmentation for the CEC condition in this experiment, confirming Hypothesis 5.

To test Hypothesis 6 we contrasted the levels of augmentation for the CE causal direction in this experiment with those observed for the EC direction in Experiment 1. The best fitting cumulative link function model had fixed main effects for causal direction and consequent with random intercepts for participants and items. This newly estimated model produced minor variations in the means for the fixed effects but the results of the simple effects comparisons were clear. The change ratings were a lot lower for the CEC condition in Experiment 2 than for the ECC condition in Experiment 1, *z*-ratio = 5.04, *p* < .0001. They were also a lot lower for the CENC condition in Experiment 2 than for the ECNC condition in Experiment 1, *z*-ratio = 5.01, *p* < .0001. These findings are not consistent with the shallow encoding hypothesis. This hypothesis must predict that the levels of augmentation should be the same in both conditions because the recoding of both pairs of rules involves a conjunction.

One might argue that these between experiment comparisons are unreliable because participants were not randomly assigned to groups in these between-subject comparisons. However, the population from which these samples were drawn was homogenous, that is, undergraduate students at UCL. Consequently, it is highly unlikely that there were other differences between these groups, other than the experimental manipulations, that could account for the large effects sizes (CEC vs ECC: *d* = 1.17 SD units, CENC vs ECNC: *d* = 1.16 SD units).

The focus of Experiment 2 has been on the shallow encoding hypothesis. However, the question arises as to whether mental models could explain these results especially if it invoked the “sole causes” condition we introduced in discussing the results of Experiment 1. Focusing on the discriminatory CE condition, (C) would be paraphrased as *if the plant is watered often AND the plant receives light*, *then it will grow*. We consider the mental models shown in Table 3, which shows all eight possibilities allowed by the three propositions in (C) and the true possibilities under four possible interpretations of the conditional that have been considered in mental models theory and which are plausible given the materials used in this experiment.

**Table 3:** Four mental model interpretations of the conditional.

	Possibilities		⊃	⊃sc	EN	≡
*Plant watered*	*Receives light*	*Grows*	*	*	*	*
*¬Plant watered*	*Receives light*	*Grows*	*	*		
*Plant watered*	*¬Receives light*	*Grows*	*	*		
*¬Plant watered*	*¬Receives light*	*Grows*	*			
*Plant watered*	*Receives light*	*¬Grows*			*	
*¬Plant watered*	*Receives light*	*¬Grows*	*	*	*	*
*Plant watered*	*¬Receives light*	*¬Grows*	*	*	*	*
*¬Plant watered*	*¬Receives light*	*¬Grows*	*	*	*	*

The left most three columns show all eight possibilities allowed by the three propositions in (C)The right most four columns show the true possibilities (*) for the material conditional (⊃), the material conditional plus sole causes (⊃sc), the enables relation (EN, see text), and the biconditional (≡).

Take the material conditional with the sole causes condition (Table 3(⊃sc)) for the consequent absent condition (CENC), Pr(*r*) = 3/6 = ½ and Pr(*r*|*p*) = 1/3 and so because 1/3–1/2 = -1/6 discounting is predicted. If we do the same calculation for the consequent (i.e., the effect) present condition (CEC), then Pr(*r*|*q*) = 2/3 and Pr(*r*|*p*, *q*) = ½, and so because ½−2/3 = -1/6, discounting is also predicted. Consequently, a model in which the conditional in the paraphrasing of the rule pairs is given by the material conditional of standard logic with a “sole cause” assumption, cannot explain the results of Experiment 2. If the “sole cause” condition is dropped (Table 3(⊃)) then mental models predicts no augmentation or discounting for CEC but it still predicts discounting for CENC, which again does not capture these results.

However, mental models theory has postulated a variety of interpretations of the conditional which usually depend on further background knowledge. This process is referred to as *pragmatic modulation* [44]. The materials in Experiment 2 for the CE condition were selected so that the causes would be interpreted as individually necessary for the effect. In mental models theory, these materials should evoke to an ENABLEs relation [44], which has been referred to as the reverse conditional [45] as it is true if the consequent is false or the antecedent is true. This is the reverse of the standard material conditional, which is true if the consequent is true or the antecedent is false. On this interpretation (Table 3(EN)), augmentation is predicted for the CENC condition but not the CEC condition, which again does not capture these results. Finally, we considered the possibility that the conditional is interpreted as the biconditional (Table 3(≡)), which is true if the consequent and antecedent are both true or both false. On this interpretation, no augmentation is predicted for either the CENC or the CEC conditions. This interpretation does not capture these results because a medium sized augmentation effect was observed for the CEC condition albeit not comparable to that seen for the EEC condition in Experiment 1. In conclusion, mental models cannot predict the results of Experiment 2.

## Conclusions

Within the limited goals that the research reported here set itself, these experiments have been successful. First, Experiment 1 replicated the results of Experiment 2 in [9] using a cumulative link function mixed model approach appropriate to these data. Using this approach allowed us to quantify, using the Bayes Factor, how much more likely CM, which uniquely predicts a main effect of causal direction, was to have generated the data than mental models theory. This support was, however, qualified because (i) no effect of consequent was observed, which CM also predicts, and (ii) the much simpler shallow encoding hypothesis could explain the pattern of results in Experiment 1. So, and second, Experiment 2 was designed to provide a critical test of the shallow encoding hypothesis. This experiment produced results inconsistent with this account. In this experiment, no augmentation effect of comparable magnitude to the diagnostic conditionals in Experiment 1 was observed for causal conditionals although in both conditions the rule pairs could be recoded using a conjunction. Moreover, there would appear to be no account of the conditional in mental models theory which could predict the results of Experiment 2. Before concluding, we address a variety of questions that might arise as a result of the research reported here.

Our results are consistent with previous work [43] and so provide convergent evidence that people implement the noisy-AND integration rule. However, the materials in [43] were all novel and participants were unlikely to have had any direct experience of the causes involved and so they had to be told that they operated conjunctively. In Experiment 2, materials that could spontaneously lead to a conjunctive interpretation were used. Individually necessary effects must be interpreted conjunctively. Our choice of materials was largely successful in replicating these previously observed effects [43] without explicitly cuing the conjunctive interpretation.

Experiment 1 was a qualified success for the causal model theory insofar as the pattern of main effects it predicts provided a better fit to the data than that predicted by mental models, although the main effect of the consequent was not observed. Mental models theory lacks the representational resources to capture the kinds of independence constraints that allow causal models to predict discounting. In this respect, causal Bayes nets is a more expressive formalism in which to capture causal relations. While there has been a recent revision of mental model theory [46], which we consider in the next paragraph, this theory has adhered to the material conditional interpretation of conditionals for most of its history. This adherence is problematic even if the equiprobability assumption is abandoned. For example, suppose it is known that *Johnny has spots* and it is asserted that *if he has chickenpox then he has spots*. Intuitively this conditional information raises the probability that Johnny has chickenpox. Moreover, this is what happens formally according to the interpretation of the conditional embodied in the causal model approach, in which the probability of the conditional is the conditional probability. However, on the material conditional interpretation, assuming equiprobability or not, the probability that Johnny has chickenpox must fall when it is learned that *if he has chickenpox then he has spots*. We have yet to test this prediction but intuitively the mental models approach gets this the wrong way round.

However, a recent radical revision of mental models theory [46] no longer commits it to the material conditional interpretation of any natural language conditional. This revision has been strongly criticised [47] and, moreover, it is easy to see that it confronts serious problems if it were to be extended to the discounting inferences which are our current topic. In the revision, the inclusive or-introduction inference, inferring “*p* or *q*” from *p*, is supposed to be logically invalid. Yet the following inference seems intuitively perfectly acceptable:

If the fuse blows or there is a power cut (*p* or *q*), then the lights go out (*r*).
The fuse blows (*p*).
Therefore, the lights go out (*r*).

Firstly, the acceptability of this inference shows how implausible it would be for pre-revision mental model theory to explain our results by claiming that the antecedent above tends to be interpreted as an exclusive disjunction. In that case, people could not generally reason, as they apparently can, in this form: “*p*, therefore *p* or *q* by inclusive or-introduction, and therefore *r* by MP” (MP = *modus ponens*, i.e., *if p*, *then q*, *p*, therefore *q*). Secondly, people could not make this inference in revised mental models theory even for inclusive disjunction, since inclusive or-introduction is supposed to be invalid. More generally, the inference “if *p* or *q* then *r*, *p*, therefore *r*” cannot be valid without the inference “*p* therefore *p* or *q*” being valid, since the validity of the former logically implies the validity of the latter via the tautology “if *p* or *q* then *p* or *q*” (see [48] for these and further problems with the revised mental models theory). Consequently, this recent revision of mental models theory is in no better shape to explain discounting effects in causal conditional inference than the pre-revision theory.

One reason why a hypothesis like shallow encoding has not been the focus of attention in the causal Bayes net literature is that there has not been much research on CBN’s as the interpretation of conditionals (for exceptions see [15, 18, 49, 50]). Accounts of violations of the Markov condition have focused more on associative, hidden cause, or mechanistic explanations [19, 51, 52, 53]. For example, for the CENC condition the sprinklers being on and rain may be negatively correlated by human intervention: there is no point in switching the sprinklers on when it is raining. So when it is raining the sprinklers are off and when the sprinklers are off it is raining. The human intervention could be characterised as an additional hidden instrumental cause in a CBN. A related positive correlation may be perceived to exist between the antecedents for the diagnostic conditionals which could explain augmentation for the ECC condition. However, there are a couple of reasons to doubt this explanation. First, the sprinkler example provides a rational explanation of why the human intervention occurs that could give rise to this association. Such an explanation is absent for rule pairs like 8 (S1A Appendix). What explanation could there be for an inverse correlation between a person’s car breaking down and them sleeping in, other than a whimsical fancy? Nonetheless, one *or* the other event will make them late for work. Second, at least for the ECC condition, associative explanations are questioned by the fact that using a different response format removes augmentation for the ECC condition [54]. In Experiment 1 in [9], difference ratings were used where, for example, in (B) participants would be asked for a rating of the probability of fever before and after they were told that someone had spots. The first rating was subtracted from the second rating to create the difference score. This procedure may remove the memory load imposed by the change rating response format. But if augmentation in the ECC condition using the change ratings is to be explained by an association between fever and spots then one would expect augmentation to also be present using the difference ratings which it was not. Our next experiments will explore further the reasons for these discrepancies.

In conclusion, discounting and augmentation inferences are a unique contribution made by the causal Bayes net approach to our understanding of human inference [11]. They have facilitated our ability to discriminate between the two main psychological theories of inference, mental models and new paradigm probabilistic approaches. The experiments testing these predictions have also thrown up other possible hypotheses such as shallow encoding which we ruled out in Experiment 2. However, while successfully dismissing one explanation we still lack an adequate explanation of the errors observed using the change ratings. Clearly, there is much further work to do in this area.

## Supporting Information

S1 Appendix Materials for Experiments 1 and 2. (DOCX)

S1 Experiment Raw data for Experiment 1. (CSV)

S2 Experiment Raw data for Experiment 2. (CSV)
