# Supplementary material for: Discounting and Augmentation in Causal Conditional Reasoning: Causal Models or Shallow Encoding?
Source: PLoS One. 2016 Dec 28;11(12):e0167741. doi: 10.1371/journal.pone.0167741 (PMC5193512; doi:10.1371/journal.pone.0167741)
Supplement: S1 Appendix — (DOCX) [file pone.0167741.s001.docx]

**Appendix: Materials**

*Appendix A: Experiment 1 Cause to Effect (CE*_OR_*)*

1. If she throws the vase, then the vase breaks (Pr(*q*|*x*): $\bar{m}$ = .88, SE = .023)

If a tennis ball hits the vase, then the vase breaks (Pr(¬*q*|¬*x*): $\bar{m}$ = .62, SE = .015)

2. If the fuse on the stereo is blown, then the stereo is off (Pr(*q*|*x*): $\bar{m}$ = .88, SE = .023)

If the stereo is unplugged, then the stereo is off (Pr(¬*q*|¬*x*): $\bar{m}$ = .51, SE = .014)

3. If she is being fired from her job, then she gets upset (Pr(*q*|*x*): $\bar{m}$ = .93, SE = .023)

If she is breaking up with her partner, then she gets upset (Pr(¬*q*|¬*x*): $\bar{m}$ = .58, SE = .014)

4. If the car has a mechanical fault, then the car stops (Pr(*q*|*x*): $\bar{m}$ = .93, SE = .012)

If the car runs out of petrol, then the car stops (Pr(¬*q*|¬*x*): $\bar{m}$ = .59, SE = .015)

5. If the battery is running out, then the watch stops

If you forget to wind the watch, then the watch stops

6. If there is an accident on the main road, then he is caught in a traffic jam

(Pr(*q*|*x*): $\bar{m}$ = .80, SE = .023)

If there are road works on the main road, then he is caught in a traffic jam

(Pr(¬*q*|¬*x*): $\bar{m}$ = .53, SE = .013)

7. If a person wants to change their career, then they book a meeting to see a careers advisor

If a person is finishing their degree, then they book a meeting with a careers advisor

8. If he sleeps in, then he is late (Pr(*q*|*x*): $\bar{m}$ = .88, SE = .015)

If his car breaks down, then he is late (Pr(¬*q*|¬*x*): $\bar{m}$ = .62, SE = .013)

9. If she has a day off from work, she is going shopping

If she is paid, she is going shopping

10. If she reads the newspapers, she learns more about world issues

If she watches the news, she learns more about world issues

*Appendix B: Experiment 1 Effect to Cause (EC*_AND_*)*

1. If she feels dizzy, then she is hungry

If her stomach is rumbling, then she is hungry

2. If it is warm outside, then it is sunny

If there are shadows, then it is sunny

3. If there are bubbles, then the water is boiling

If there is steam, then the water is boiling

4. If I am shivering, then I am cold

If my hairs are raised, then I am cold

5. If there are puddles in the road, then it has been raining

If my clothes are wet, then it has been raining

6. If the food is piping hot, then the food is cooked

If the food is golden brown, then the food is cooked

7. If your colleague has time off work, then he is still ill

If your colleague visits the Dr, then he is still ill

*Appendix C: Experiment 2 Cause to Effect Conjunctive Interpretation (*CE_AND_*)*

1. If she needs a break from work, then she books a cruise (Pr(*q*|*x*): $\bar{m}$ = .84, SE = .016)

If she wants to travel abroad, then she books a cruise (Pr(¬*q*|¬*x*): $\bar{m}$ = .67, SE = .016)

2. If she shares her toys, then the teacher gives her a good report

(Pr(*q*|*x*): $\bar{m}$ = .66, SE = .012)

If she speaks politely, then the teacher gives her a good report

(Pr(¬*q*|¬*x*): $\bar{m}$ = .70, SE = .020)

3. If he is tactical, then he wins the fight (Pr(*q*|*x*): $\bar{m}$ = .80, SE = .016)

If he is determined, then he wins the fight (Pr(¬*q*|¬*x*): $\bar{m}$ = .63, SE = .016)

4. If the plant is watered often, then it grows well (Pr(*q*|*x*): $\bar{m}$ = .74, SE = .016)

If the plant receives light, then it grows well (Pr(¬*q*|¬*x*): $\bar{m}$ = .79, SE = .016)

5. If her room is clean, then she is allowed to go out (Pr(*q*|*x*): $\bar{m}$ = .71, SE = .018)

If her homework is complete, then she is allowed to go out

(Pr(¬*q*|¬*x*): $\bar{m}$ = .77, SE = .021)

6. If he has valid insurance, then he drives the car

If the car has a valid MOT, then he drives the car

*Appendix D: Experiment 2 Effect to Cause Disjunctive Interpretation (*EC_OR_*)*

1. If the participant wears glasses, then their vision is poor

If the participant wears contact lenses, then their vision is poor

2. If he goes to the local shop, then the milk has run out

If he has a black coffee, then the milk has run out

3. If she is ordering coffee, then she fancies a hot drink

If she is ordering tea, then she fancies a hot drink

4. If the food is undercooked, then the oven is faulty

If the food is burnt, then the oven is faulty

5. If the occupier is looking to buy a new property, then she wants to move

If the occupier is looking to rent a new property, then she wants to move

6. If she is renting DVDs for a movie night, then she is in the mood to watch a film

If she is going to the cinema, then she is in the mood to watch a film
